# Arbuscular Mycorrhizal Fungi (*Rhizophagus clarus*) and Rhizobacteria (*Bacillus subtilis*) Can Improve the Clonal Propagation and Development of Teak for Commercial Plantings

**DOI:** 10.3389/fpls.2021.628769

**Published:** 2021-07-02

**Authors:** Flávia Sampaio Alexandre, Larissa Venturini Della Flora, Ivanildo Guilherme Henrique, Daniely Camila da Silva, Andreza Pereira Mercedes, Aline Cardoso Silva, Afonso Silva de Oliveira, Mariane Patrícia Bondespacho da Silva, Bruna Patrícia Formelh Ronning, Douglas Rafael Dreher, Bárbara Gionco Cano, Matheus Felipe de Lima Andreata, Joamir Barbosa Filho, Eva Roseane Santos, Fausto Hissashi Takisawa, Rafael Ferreira Alfenas, Galdino Andrade, Martha Viviana Torres Cely

**Affiliations:** ^1^Laboratorio de Microbiologia do Solo e Biotecnologia Agrícola, Universidade Federal de Mato Grosso, Sinop, Brazil; ^2^Laboratório de Ecologia Microbiana, Departamento de Microbiologia, Universidade Estadual de Londrina, Londrina, Brazil; ^3^TRC, Teak Resources Company, Cáceres, Brazil; ^4^Laboratório de Patologia Florestal, Universidade Federal de Viçosa, Viçosa, Brazil

**Keywords:** forestry, nursery, soil microbiology, *Tectona grandis*, wood production

## Abstract

The *Tectona grandis* L.f. (teak) is an important forest species with high economy value in Asia, Africa, and Latin America. In Latin America, Brazil is one of the countries with the most cultivated areas. The cultivation of teak turns out to be challenging because of its high nutritional demand and the need for seedling production by clonal propagation that includes about 90 days in the nursery phase. The optimization of seedling production is necessary for better results in the nursery and to enhance growth in the field. In this way, the well-known advantage of using microorganisms that promote plant development appears as a potential biotechnological approach to be explored and for the implantation of new areas of wood production. In this study, the inoculation of *Bacillus subtilis* as plant growth-promoting rhizobacteria (PGPR) was evaluated, and *Rhizophagus clarus*, an arbuscular mycorrhizal fungus (AMF), and the co-inoculation of these microorganisms in the teak seedling production phase can improve the development of commercial plantations under field conditions. Experiments were carried out under greenhouse and field conditions to evaluate four treatments based on the substrate inoculation of the seedlings. Treatments consisted of a non-inoculated control, PGPR inoculation, AMF inoculation, and PGPR + AMF inoculation. The results of the biometric evaluation of seedlings in the greenhouse showed that there was a significant difference in AMF inoculation and PGPR + AMF inoculation in terms of the specific root length and root density treatments, there was also a positive correlation between these two treatments and the absorption of some nutrients, such as P, N, K, Mg, Cu, Mn, and Zn. This response led to an increase between 4.75 and 11.04% in the field growth rate.

## Introduction

The forestry sector has become increasingly important for the Brazilian gross domestic product (GDP), having 1.3% share in 2018, reaching a total revenue of 86.6 billion. The cultivation of trees for industrial purposes is a renewable source of raw material for almost 5,000 products, such as sawn wood, paper, cellulose, floors, panels, and charcoal, being an important weapon in the recovery of degraded areas and protection of habitats (IBÁ, 2019).

Being one of the main nobles manufactured in the world, teak (*Tectona grandis* L.f.) is a tree species in the Lamiaceae family, native to the Asian continent, initiated throughout tropical Asia, as well as tropical Africa, Latin America, and the Caribbean. It is characterized by its soft color, fine grain, and durability, and it is commonly used for products of high commercial value, such as furniture, shipbuilding, and decorative construction components (Pandey and Brown, 2000). In Brazil, ~93,957 ha of planted areas of this species were registered in 2018 (IBÁ, 2019).

A limiting factor for its cultivation is the low germination rate of seeds and their sensitivity to storage, with genetic improvement being one of the main alternatives to this problem, bringing long-term results (Schuhli and Paludzyszyn Filho, 2010). In this scenario, the clonal production of selected trees has been boosted. The mini-cutting technique can provide, in the short term, homogeneous seedlings with desired characteristics, increase the number of rooted mini-cuttings, and improve the root system, directly influencing the performance of seedlings in the field (Meza et al., 2015).

The inoculation of plant growth-promoting rhizobacteria (PGPR) during seedling production has already proved efficient, generating healthier plants with gains in biomass and nutrient content (Rodrigues et al., 2018; Raghu et al., 2020). Moreover, the response of teak to different and selected microbial consortiums, such as *Ambispora leptoticha, Azotobacter chroococcum*, and *Trichoderma harzianum* has already been tested (Raghu et al., 2020). Among the PGPR, *Bacillus* is one of the most important genera in soil, and its ability to generate spores increases its survival and competitiveness in adverse conditions. The genus assists plants in their defense against attacks by pathogens and increases their tolerance to stress (Hashem et al., 2016).

Arbuscular mycorrhizal fungi (AMF) also improve plant growth (Akinrinlola et al., 2018). AMF are able to promote growth, and they are resistant to biotic and abiotic stresses, because of greater absorption of nutrients, with emphasis on phosphorus (P) (Smith and Read, 2008). They increase the formation of soil aggregates through the production of glomalin, and they are also important regulators of soil stability and quality (Rillig and Mummey, 2006). A greenhouse study demonstrated the beneficial effect of AMF inoculation on teak seedlings, with greater growth of aerial parts and roots, content of nutrients, such as potassium (K) and sulfur (S), and better efficiency in the use of nutrients, such as P (Rodrigues et al., 2018). Another study conducted to examine the efficacy of three native species of AMF (*Funneliformis mosseae, Glomus intradices*, and *Glomus proliferum*) on the growth and seedling quality of *T grandis* showed that mycorrhizal symbiosis significantly improved seedling growth and physiological parameters, proving that this technology has a potential to reduce the nursery period and that it increases the quality of produced seedlings, resulting in considerable economic gains (Ajeesh et al., 2017).

The inoculation of AMF and *Bacillus* species and the co-inoculation of these microorganisms have already been proven as efficient methods to increase plant growth by several researchers (Medina et al., 2003; Marulanda-Aguirre et al., 2007; Alam et al., 2011; Awasthi et al., 2011). *B subtilis* is sold commercially (Biobaci®), and *R clarus* is in the process of launching the commercial product (Andrade and Cely, 2019), facilitating the implantation of these microorganisms in the operational routine of companies for the production of teak seedlings. Thus, this study aims to demonstrate the effect of AMF *R clarus* and PGPR *B subtilis*, their co-inoculation interaction in the nursery phase, and their potential to improve the growth process of *T grandis* under field conditions.

## Materials and Methods

### Seedling Experiments

The experiment was carried out in the Teak Resources Company (TRC) seedling nursery, located in Jangada city, MT, Brazil from September 2016 to January 2017. The principal commercial clone of the company was multiplied by mini-cutting systems from clonal garden. Microorganisms were incorporated in the substrate of pine bark (Mec Plant - Florestal 2) fertilized with Osmocote Mini Prill-3M (N 19%, P_2_O_5_ 6%, and K_2_O 10%) −8 g + PG Mix (N 14%, P_2_O_5_ 16%, and K_2_O 18%) −5 g/kg of substrate. Before planting, mini-cuttings were dipped in indole butyric acid (IBA) (500 mg L^−1^) and then planted in tubes of 53 cm^3^. The treatments presented in Table 1 are considered. Two types of microorganisms were inoculated: AMF *R clarus*, with a concentration of 200 propagules/ml multiplied *in vitro* system according to the patent application: BR 102019008109-0 A2 of 22/04/2019 (Andrade and Cely, 2019) and commercial product Biobaci® containing viable cells (1 × 10^8^ CFU/ml) of PGPR *B subtilis*. In total, 2,112 seedlings were produced (528 seedlings per treatment). A completely randomized design was chosen under greenhouse conditions.

**Table 1:** Treatments and doses used for incorporation into the substrate of microorganisms *Rhizophagus clarus* and *Bacillus subtilis* in the production of *Tectona grandis* seedlings.

**Treatment**	**Description**
T1	Control (substrate without microorganisms)
T2	PGPR (*Bacillus subtilis*)- 50 mL/L of substrate
T3	AMF (*Rhizophagus clarus*) - 15 g/L of substrate
T4	PGPR + AMF(*Bacillus subtilis*- 50 mL + *Rhizophagus clarus*- 15 g/L of substrate)

The mini-cuttings were kept in an air-conditioned greenhouse with a transparent polyethylene cover and 50% shadowing fabric for 30 days. Parameters, namely, relative humidity (RH > 80%), temperature (35–40°C), and irrigation were controlled *via* nebulization. In the 1st week, the frequency of irrigation was 10 s each 15 min; and in the following weeks, the frequency was 20 s each 40 min (24 L/h/micro sprinkler). Subsequently, they were placed in the shade house with 50% shadowing fabric and 4 h of irrigation for 15 min, for acclimatization until they reached 60 days. During this phase, the seedlings were fertigated once a day with 600 mg. L^−1^ of the following substances: MgSO_4_ 7H_2_O, Ca (NO_3_)_2_, and NH_4_NO_3_ each; 800 mg. L^−1^ of NH_4_H_2_PO_4_, and 400 mg. L^−1^ of KNO_3._ After this period, the seedlings went full sun until they completed 90 days. The frequency of irrigation was every 6 h for 10 min (810 L/h/micro sprinkler), and fertigation was performed twice a week with the same nutrient solution mentioned above.

### Data Collected in Nursery Phase

Seedling survival was evaluated 30, 60, and 90 days after planting. Ninety days after planting, 20 seedlings per treatment were collected for biometric analysis in the laboratory. At this time, height = H (cm), stem diameter = SD (mm), dry shoot biomass = SB (g), root biomass = RB (g) and biomass ratio of the root/shoot = RB/SB (g) were evaluated. The remaining seedlings were intended for field experiments.

Ten root seedlings samples per treatment were also collected to evaluate mycorrhizal colonization. The percentage of mycorrhizal colonization was estimated by the grid-line method (Giovannetti and Mosse, 1980) after staining roots with Trypan blue (0.05%) (Phillips and Hayman, 1970). Total root length = TRL (cm), specific root length = SRL (cm g^−1^), and root density = RD (g cm^−3^) were also evaluated at this stage (Ryser and Lambers, 1995). Twenty seedling samples per treatment were separated for macro and micronutrient foliar analysis; and for this, the following methods were used sulfuric digestion and quantification by titration after semi-micro Kjeldahl distillation (N), nitric-perchloric digestion (P, K, Ca, Mg, S, Cu, Fe, Mn, Zn, Na, and Ni), incineration (B, Co, and Mo), agitation (Cl). Sample reading was made by inductively coupled plasma-optical emission spectrometry (ICP-OES) with a Thermo Scientific ICAP 7600 spectrometer (Thermo Fisher Scientific, Waltham, Massachusetts, US).

### Field Experiments

The field experiments were started in February 2017. Two experimental areas were used. Area I is located in Cáceres city, state of Mato Grosso (MT), Brazil (16°8′1.75″ S and 58°31′1.77″ W) and has tropical savanna climate (Aw) (Köppen, 1936) and soil classification We-Eutric Planosols (FAO, 1994). Area II is located in Santa Maria das Barreiras city, state of Pará (PA), Brazil (8°43′1.11″ S and 50°29′10.76″ W) and has tropical savanna climate (Aw) (Köppen, 1936) and soil classification Ao-Orthic Acrisols (FAO, 1994). Soil samples were collected in Area I and Area II for physicochemical analysis at depths of 0–20 and 20–40 cm. The analysis showed the following results: Area I depth 0–20 cm: pH (CaCl_2_) 5.1, Al^+3^ 0 cmol_c_ dm^−3^, H + Al 2.48 cmol_c_ dm^−3^, P 3.5 mg dm^−3^, K^+^ 30.3 mg dm^−3^; Ca^+2^ 1.5 cmol_c_ dm^−3^, and Mg^+2^ 0.58 cmol_c_ dm^−3^. Depth 20–40 cm with pH (CaCl_2_) 4.6, Al^+3^ 0.28 cmol_c_ dm^−3^, H + Al 2.25 cmol_c_ dm^−3^, P.3 mg dm^−3^, K^+^ 16.2 mg dm^−3^; Ca^+2^ 0.90 cmol_c_ dm^−3^, and Mg^+2^ 0.37 cmol_c_ dm^−3^. Area II depth 0–20 cm: pH (CaCl_2_) 4.5, Al^+3^ 0.2 cmol_c_ dm^−3^, H + Al 2.2 cmol_c_ dm^−3^, P 1.8 mg dm^−3^, K^+^ 24.6 mg dm^−3^, Ca^+2^ 1.2 cmol_c_ dm^−3^, and Mg^+2^ 0.4 cmol_c_ dm^−3^. Depth 20–40 cm with pH (CaCl_2_) 4.4, Al^+3^ 0.2 cmol_c_ dm^−3^, P.5 mg dm^−3^, K^+^ 19.5 mg dm^−3^, Ca^+2^ 0.80 cmol_c_ dm^−3^; and Mg^+2^ 0.3 cmol_c_ dm^−3^. pH (CaCl_2_) was evaluated in a 0.01 M chloride solution, in the proportion 1:2.5 (soil: CaCl_2_). P and K^+^ were extracted with a 0.05 N HCl and 0.025 N H_2_SO_4_ solution (Mehlich I). Ca^+2^, Mg^+2^, and Al^+3^ were extracted with a 1 N potassium chloride solution.

**Figure 1:**
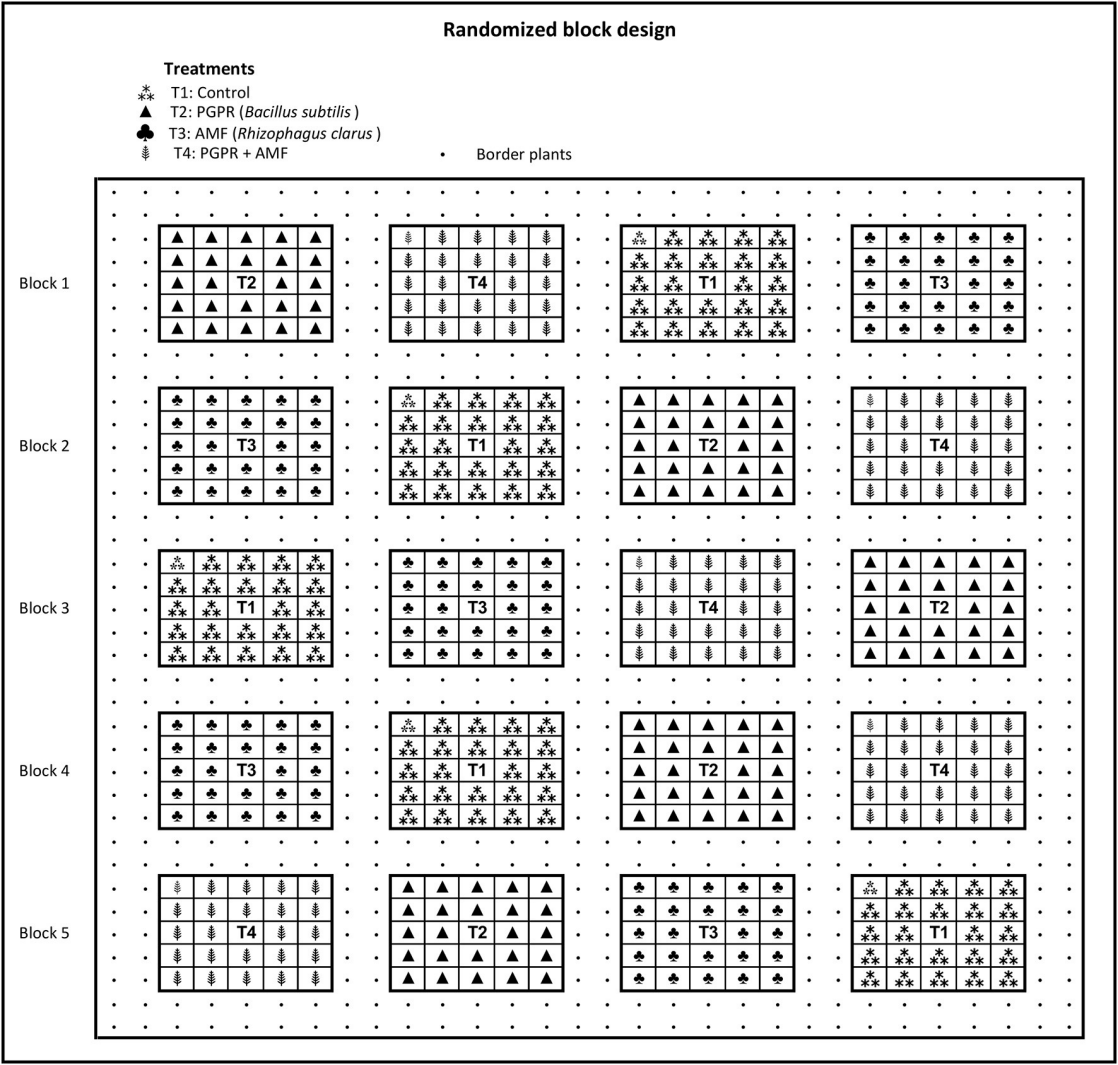
Randomized block design of field experiments with *Tectona grandis* in states Mato Grosso and Pará, Brazil.

These experiments were carried out in a randomized block design. Five blocks were installed, and within each block four plots of 5 × 5 plants (25 plants per plot) were used. Each plot corresponds to one of the treatments defined in the nursery phase (Table 1), separated by two border plants (Figure 1). Plant spacing was 3 × 4 m. Total experimental area was 1.33 ha of planting including border plants. For the implantation and conduction of planting, the standard routine of the company was followed.

### Data Collected in Field

Plant survival was evaluated 30, 60, and 90 days after planting in the field. Data collection was made 2 and 3 years after planting. The data collected in this time were: height = H (m) and diameter at breast height = DBH (cm), tree volumes = V (dm^3^) and were calculated with these data, using the equation [π * (DBH^2^/4) * H * Ff]. The artificial form factor = Ff = 0.63 was used, the average value indicated for young teak plantation, between 1 and 3 years old (Carneiro et al., 2018). Additionally, the mean annual increment = MAI (dm^3^ year^−1^) and the current annual increment = CAI (dm3) were estimated.

### Statistical Analysis

To evaluate the effects of treatments on seedling survival, we performed generalized linear models (GLMs) analysis. For models, the treatments (T1, T2, T3, and T4) were used as predictors and the time as an independent factor. The statistical significance (p) of each predictor is based on chi-square tests to assess the significance of the change in deviance for each predictor. For model interpretation, the odds ratio (OR) and its confidence interval were calculated.

Statistical analyses of AMF root colonization were performed using the Friedman test at a significance level of *p* ≤ 0.05. Plant growth parameters in the seedling experiments were analyzed by analysis of variance (ANOVA) and the Tukey test (*p* ≤ 0.05). Principal component analysis (PCA) was also performed for nutrient content. Analyses of field data at 2 and 3 years after planting were performed by factorial ANOVA. Factor A: treatment. Factor B: block. For comparison of means, the Tukey test (*p* ≤ 0.05) was performed. All statistical analyses in this research were performed using the R software (R Core Team, 2020).

## Results

### Seedling Test

The results of GLMs based on the patterns of seedling survival in the nursery phase revealed significant effects of treatments as predictors for this parameter. That is, the treatments can be used to explain the survival pattern in this experiment, being that all models were statistically significant (*p* < 0.001). The OR showed better survival for seedlings of teak when inoculated with PGPR (*B subtilis*) and AMF (*R clarus*) (Table 2).

**Table 2:** Summary of the general linear models (GLMs) of *Tectona grandis* seedling survival at 30, 60, and 90 days in nursery.

**Predictor**	**df**	**Estimate**	***X_**2**_***	***P***	**OR**	**Confidence interval**	
						**25%**	**95%**
T1	1	1.090	40.75	1.7^−10^^*^	2.97	2.12	4.15
T2	1	1.732	73.70	2.2^−16^^*^	5.65^**^	3.80	8.38
T3	1	1.247	58.11	2.4^−14^^*^	3.48^**^	2.52	4.79
T4	1	0.798	19.06	1.2^−05^^*^	2.22	1.55	3.18

* *Significant predictors at (p ≤ 0.001)*.

** *Highest odds ratio for survival*.

The mycorrhizal colonization in the treatments inoculated with *R clarus* (AMF) was just over 60%. In general, bacterial inoculation with PGPR did not influence the rate of colonization of teak roots by mycorrhiza (Table 3).

**Table 3:** Percentage of mycorrhizal colonization of *Tectona grandis* seedling at 90 days.

**Treatment**	**Mycorrhizal colonization (%)**
T1 – Control	0.0^b^
T2 – PGPR	0.0^b^
T3 – AMF	63.4^a^
T4 – PGPR+ AMF	61.8^a^

*Treatments with the same letter are not significantly different by Friedman test (p ≤ 0.05)*.

**Figure 2:**
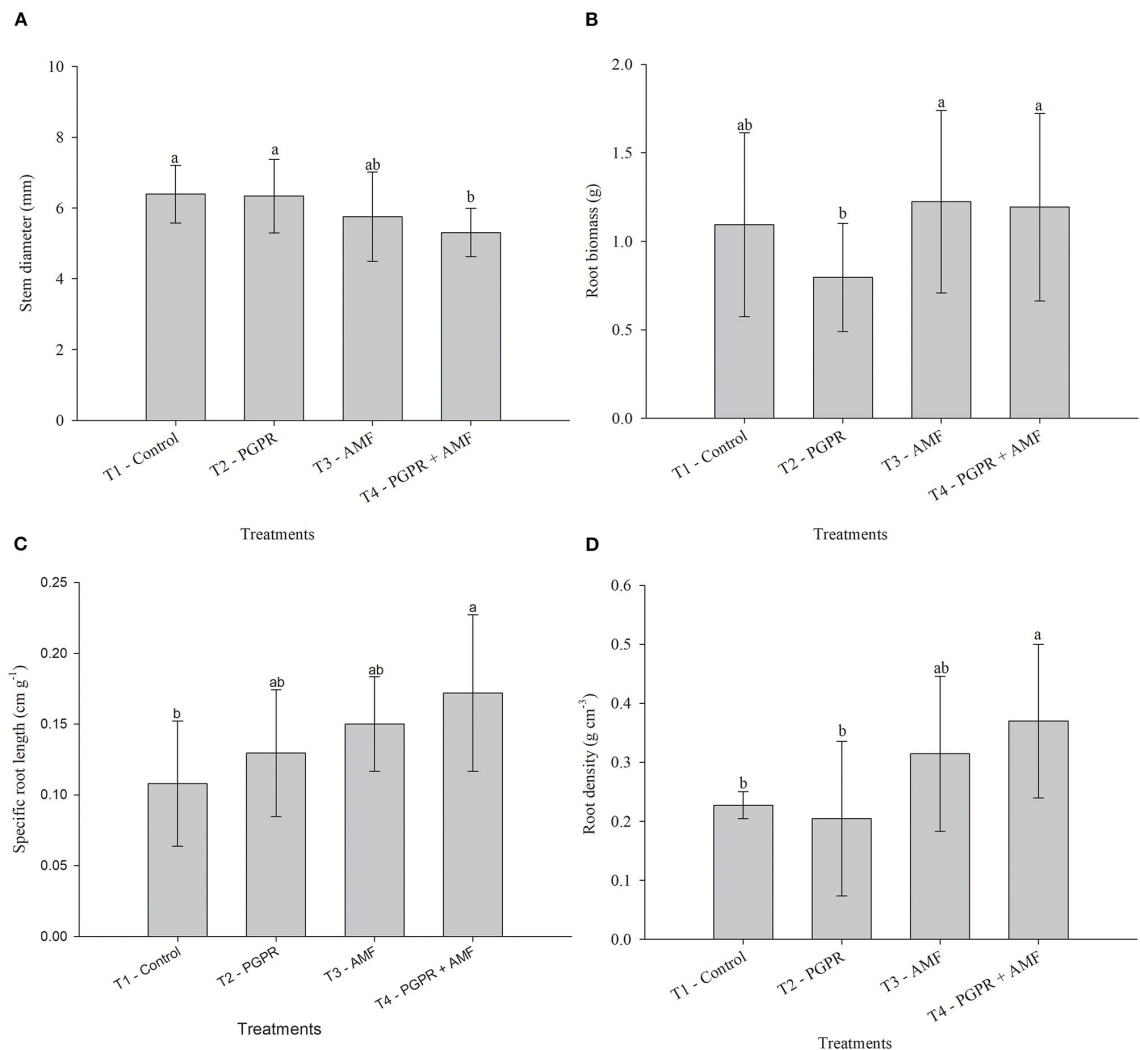
Effect on seedling growth of *Tectona grandis* at 90 days. **(A)** Stem diameter. **(B)** Root biomass. **(C)** Specific root length. **(D)** Root density (*n* = 20). Bars followed by the same letter do not differ by Turkey test (*p* ≤ 0.05). Bars represent standard deviation of means.

In the seedling growth evaluation, the treatments have a significant effect on stem diameter, root biomass, specific root length, and root density (*p* ≤ 0.05) (Table 4). For stem diameter, the treatments with microorganisms PGPR and AMF were statistically equal to the control, only PGPR + AMF was lower than the control treatment (Figure 2A). AMF inoculation resulted in a significant decrease in root biomass, while the other two microbial treatments did not affect this variable (Figure 2B). The low development of stem diameter in PGPR + AMF was compensated by differentiate response in root system inoculation that showed gains in specific root length and root density (Figures 2C,D).

**Table 4:** Analysis of variance (ANOVA) for treatments of seedling growth parameters of *Tectona grandis* at 90 days after mini-cuttings planting.

**Variables**	**df**	***F***	***p*-value**	**Error**
**ANOVA**				
H	3	0.834	0.48000	31.30
SD	3	5.588	0.00162^*^	0.951
SB	3	2.333	0.08070	0.870
RB	3	3.345	0.02350^*^	0.228
RB/SB	3	1.974	0.13500	0.096
TRL	3	0.670	0.57600	369.2
SRL	3	3.631	0.02190^*^	0.002
RD	3	4.520	0.00864^*^	0.013

*H (cm), Height; SD (mm), stem diameter; SB (g), dry shoot biomass; RB (g), root biomass; RB/SB (g), biomass ratio of the root/shoot; TRL (cm), total root length; SRL (cm g^−1^), specific root length; RD (g cm^−3^), root density*.

* *Significant difference (p ≤ 0.05)*.

**Figure 3:**
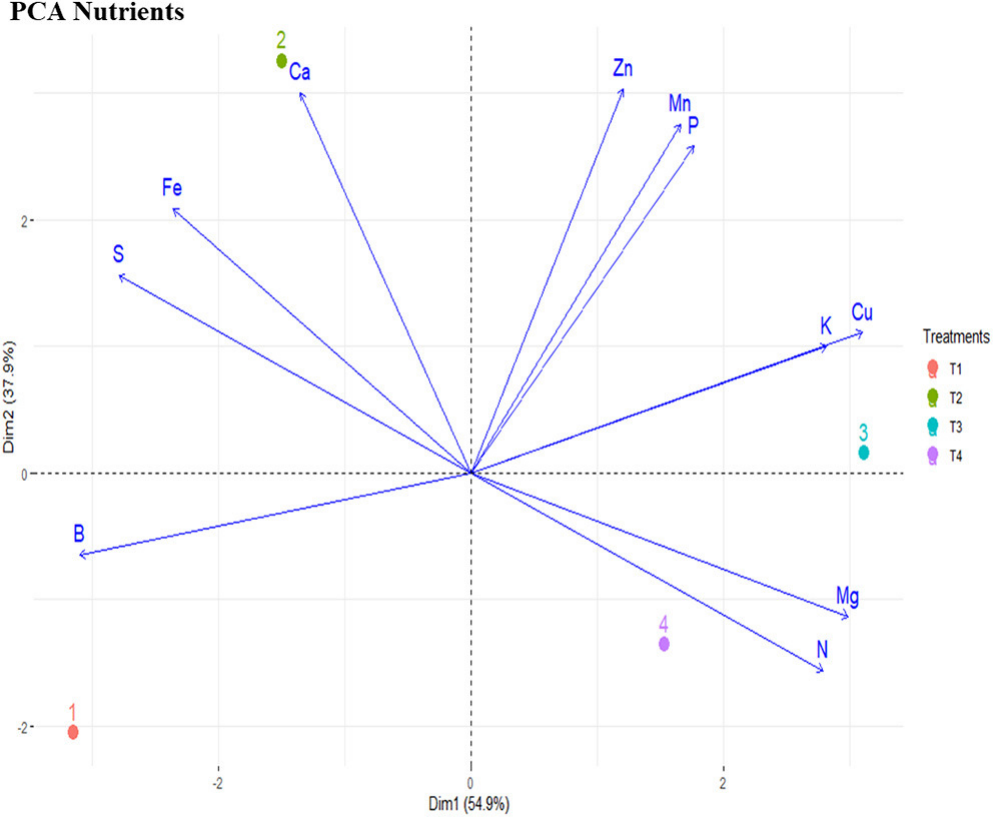
Principal component analysis for nutrients on *Tectona grandis* seedling at 90 days. T1, Control; T2, PGPR *Bacillus subtilis*; T3, AMF *Rhizophagus clarus*; T4, PGPR + AMF.

In the PCA, 92.8% of the variability of nutrients data were represented in components one and two. In the first dimension, representing 54.9% of the data variability, macronutrients nitrogen (N), phosphorus (P), and potassium (K) were positively related with treatments T3 and T4 (AMF inoculation and PGPR + AMF inoculation), as well as micronutrients magnesium (Mg), copper (Cu), manganese (Mn), and Zinc (Zn). In the second dimension, representing 37.9% of the data variability, calcium (Ca), iron (Fe), and sulfur (S) data were associated with treatment T2 (PGPR). The control treatment was allocated far from the eigenvalues of the nutrients, showing that the treatment did not interact with the nutritional data (Figure 3).

### Field Test

In the field, seedling survival is not explained by the treatments as predictors in GLM in the Mato Grosso area. On the other hand, in Pará, treatment with *B subtilis* (T2) showed 96% of seedling survival and was considered a predictor for this parameter with GLM statistically significant (*p* < 0.05). This treatment showed an OR of 12.55 (confidence interval of 2.5–95%/1.49–105.45) (Table 5).

**Table 5:** Summary of the general linear models (GLMs) of *Tectona grandis* seedling survival at 30, 60, and 90 days in field.

**Predictor**	**df**	**Estimate**	***X_**2**_***	***p***
**Mato Grosso**				
T1	1	1.398	1.29	0.2555
T2	1	14.27	7e-04	0.9787
T3	1	14.27	7e-04	0.9787
T4	1	14.27	7e-04	0.9787
**Pará**				
T1	1	16.31	8^−04^	0.9781
T2	1	2.53	5.42	0.0198^*^
T3	1	16.29	5^−04^	0.9821
T4	1	16.31	8^−04^	0.9781

* *Significant predictors at (p ≤ 0.05)*.

The tree volume (V) and mean annual increment (MAI) at 2 years after planting in Mato Grosso showed statistical differences between treatments, block, and the interaction between treatment × block. Treatments T1, T2, and T4 were significantly equal, and treatment T3 was statistically lower in both variables analyzed. The plants in block 2 performed better than the plants in blocks 3 and 4. The interaction of the treatment and blocking factors was significant but did not show any clear pattern (Table 6).

**Table 6:** Tree volume and mean annual increment at 2 years old in plants of *Tectona grandis* in Mato Grosso state, Brazil.

**Factor**		**V (dm**^****3****^**)**		**MAI (dm**^****3****^ **year**^****−1****^**)**		
	**df**	***F***	***p*-value**	***F***	***p*-value**	
**ANOVA - Mato Grosso, 2 years old**						
Treatment (A)	3	11.37	0.0000^*^	11.351	0.0000^*^	
Block (B)	4	6.13	0.0000^*^	6.131	0.0000^*^	
A × B	12	15.09	0.0000^*^	15.074	0.0000^*^	
Error		0.224		0.056		
**Treatment**			**V (dm**^**3**^**)**			**MAI (dm**^**3**^ **year**^**−1**^**)**
**Tukey test of treatment**						
T1 – Control			0.96 ± 0.46^a^			0.48 ± 0.23^a^
T2 – PGPR			0.92 ± 0.55^a^			0.46 ± 0.27^a^
T3 – AMF			0.70 ± 0.33^b^			0.35 ± 0.16^b^
T4 – PGPR + AMF			1.04 ± 0.79^a^			0.52 ± 0.40^a^
**Block**			**V (dm**^**3**^**)**			**MAI (dm**^**3**^ **year**^**−1**^**)**
**Tukey test of block**						
1			0.98 ± 0.91^ab^			0.49 ± 0.45^ab^
2			1.06 ± 0.58^a^			0.53 ± 0.29^a^
3			0.82 ± 0.35^bc^			0.41 ± 0.18^bc^
4			0.77 ± 0.35^c^			0.38 ± 0.17^c^
5			0.89 ± 0.42^abc^			0.45 ± 0.21^abc^
**Block**	**T1 - Control**		**T2 – PGPR**	**T3 – AMF**		**T4 - PGPR + AMF**
**Tukey test of A × B V (dm**^**3**^**)**						
1	0.86 ± 0.44^cd^		0.56 ± 0.24^d^	0.55 ± 0.23^d^		1.99 ± 1.30^a^
2	1.16 ± 0.49^bc^		1.53 ± 0.73^ab^	0.70 ± 0.29^cd^		0.85 ± 0.31^cd^
3	0.73 ± 0.36^cd^		0.89 ± 0.35^cd^	0.81 ± 0.32^cd^		0.85 ± 0.37^cd^
4	0.92 ± 0.42^cd^		0.80 ± 0.30^cd^	0.57 ± 0.25^d^		0.81 ± 0.33^cd^
5	1.13 ± 0.48^bc^		0.80 ± 0.42^cd^	0.90 ± 0.40^cd^		0.73 ± 0.24^cd^
**MAI (dm**^**3**^ **year**^**−1**^**)**						
1	0.43 ± 0.22^cd^		0.28 ± 0.12^d^	0.28 ± 0.12^d^		0.99 ± 0.65^a^
2	0.58 ± 0.25^bc^		0.77 ± 0.37^ab^	0.35 ± 0.15^cd^		0.42 ± 0.16^cd^
3	0.36 ± 0.18^cd^		0.44 ± 0.18^cd^	0.41 ± 0.16^cd^		0.42 ± 0.19^cd^
4	0.48 ± 0.21^cd^		0.40 ± 0.15^cd^	0.28 ± 0.13^d^		0.40 ± 0.16^cd^
5	0.57 ± 0.24^bc^		0.40 ± 0.21^cd^	0.45 ± 0.20^cd^		0.36 ± 0.12^cd^

* *Significant difference (p ≤ 0.05). Same letter does not differ by the Tukey test (p ≤ 0.05)*.

At 3 years after planting in Mato Grosso, statistical differences between treatments, block, and the interaction between treatment × block for V, MAI, and CAI were observed. The results were similar to those presented at 2 years for V and MAI and for CAI (Table 7).

**Table 7:** Tree volume, mean annual increment, and current annual increment at 3 years old in plants of *Tectona grandis* in Mato Grosso state, Brazil.

**Factor**		**V (dm**^****3****^**)**		**MAI (dm**^****3****^ **year**^****−1****^**)**		**CAI (dm**^****3****^**)**	
	**df**	***F***	***p*-value**	***F***	***p*-value**	***F***	***p*-value**
**ANOVA - Mato Grosso, 3 years old**							
Treatment (A)	3	13.464	0.0000^*^	13.466	0.0000^*^	12.906	0.0000^*^
Block (B)	4	4.615	0.0012^*^	4.614	0.0012^*^	4.411	0.0017^*^
A × B	12	10.526	0.0000^*^	10.529	0.0000^*^	9.790	0.0000^*^
Error		41.2		4.58		37.4	
**Treatment**		**V (dm**^**3**^**)**		**MAI (dm**^**3**^ **year**^**−1**^**)**		**CAI (dm**^**3**^**)**	
**Tukey test Treatment**							
T1 – Control		23.62 ±6.80^a^		7.87 ± 2.27^a^		22.66 ± 6.44^a^	
T2 – PGPR		22.85 ± 7.82^a^		7.62 ± 2.61^a^		21.93 ±7.40^a^	
T3 – AMF		19.65 ± 5.56^b^		6.55 ± 1.85^b^		18.95 ± 5.33^b^	
T4 - PGPR + AMF		24.57 ± 8.40^a^		8.19 ± 2.80^a^		23.53 ± 7.85^a^	
**Block**		**V (dm**^**3**^**)**		**MAI (dm**^**3**^ **year**^**−1**^**)**		**CAI (dm**^**3**^**)**	
**Tukey test of block**							
1		23.85 ± 8.92^ab^		7.95 ± 2.97^ab^		22.87 ± 8.23^ab^	
2		24.38 ± 8.30^a^		8.13 ± 2.77^a^		23.31 ± 7.82^a^	
3		21.27 ± 6.36^c^		7.09 ± 2.12^c^		20.45 ± 6.12^c^	
4		22.28 ± 6.86^abc^		7.43 ± 2.29^abc^		21.51 ± 6.64^abc^	
5		21.53 ± 5.90^bc^		7.18 ± 1.97^bc^		20.64 ± 5.60^bc^	
**Block**	**T1 – Control**		**T2 – PGPR**		**T3 – AMF**		**T4 – PGPR + AMF**
**Tukey test of A × B V (dm**^**3**^**)**							
1	23.55 ± 6.88^cde^		20.03 ± 4.28^de^		18.20 ± 5.80^e^		34.02 ±8.61^a^	
2	25.17 ± 6.17^bcd^		30.85 ± 8.94^ab^		19.40 ± 5.58^de^		21.90 ± 7.62^cde^	
3	21.11 ± 6.86^cde^		22.92 ± 7.42^cde^		20.66 ± 4.96^cde^		20.37 ± 5.99^cde^	
4	22.78 ± 7.12^cde^		20.77 ± 6.29^cde^		19.06 ± 5.49^de^		26.85 ± 6.27^bc^	
5	25.38 ± 6.57^bcd^		19.60 ± 5.61^de^		20.99 ± 5.87^cde^		20.09 ± 3.46^de^	
**MAI (dm**^**3**^ **year**^**−1**^**)**							
1	7.85± 2.29^cde^		6.68± 1.43^de^		6.07± 1.83^e^		11.34± 2.87^a^	
2	8.39 ± 2.06^bcd^		10.28 ± 2.98^ab^		6.47 ± 1.86^de^		7.30 ± 2.54^cde^	
3	7.04 ± 2.29^cde^		7.64 ± 2.47^cde^		6.89 ± 1.65^cde^		6.79 ± 2.00^cde^	
4	7.59 ± 2.37^cde^		6.92 ± 2.10^cde^		6.35 ± 1.83^de^		8.95 ± 2.09^bc^	
5	8.46 ± 2.19^bcd^		6.53 ± 1.87^de^		7.00 ± 1.96^cde^		6.70 ± 1.15^de^	
**CAI (dm**^**3**^**)**							
1	22.69 ± 6.53^cde^		19.47 ± 4.27^de^		17.65 ± 5.69^e^		32.03 ± 7.71^a^	
2	24.01 ± 5.78^bcd^		29.32 ± 8.32^ab^		18.70 ± 5.35^de^		21.05 ± 7.78^cde^	
3	20.38 ± 6.57^cde^		22.03 ± 7.15^cde^		19.85 ± 4.78^cde^		19.52 ± 5.73^de^	
4	21.86 ± 6.85^cde^		19.97 ± 6.09^cde^		18.50 ± 5.39^de^		26.04 ± 6.12^abc^	
5	24.24 ± 6.19^bcd^		18.80 ± 5.42^de^		20.09 ± 5.53^cde^		19.36 ± 3.33^de^	

* *Significant difference (p ≤ 0.05). Same letter does not differ by the Tukey test (p ≤ 0.05)*.

In Pará, the V and MAI showed statistical differences between treatments, block, and the interaction between treatment × block at 2 years. The T2 andT4 treatments presented the best results and were statistically superior to T1 and T3. The plants in blocks 1, 2, and 3 performed better than the plants in blocks 4 and 5. The interaction of the treatment and blocking factors was significant but did not show any clear pattern (Table 8).

**Table 8:** Tree volume and mean annual increment at 2 years old in plants of *Tectona grandis* in Pará state, Brazil.

**Factor**		**V (dm**^****3****^**)**		**MAI (dm**^****3****^ **year**^****−1****^**)**	
	**df**	***F***	***p*-value**	***F***	***p*-value**
**ANOVA - Pará, 2 years old**					
Treatment (A)	3	2.929	0.0334^*^	2.928	0.0335^*^
Block (B)	4	36.842	0.0000^*^	36.843	0.0000^*^
A × B	12	6.807	0.0000^*^	6.808	0.0000^*^
Error		36.4		9.1	
**Treatment**		**V (dm**^**3**^**)**			**MAI (dm**^**3**^ **year**^**−1**^**)**
**Tukey test**						
T1 - Control		16.50 ± 7.37^b^			8.25 ± 3.68^b^
T2 - PGPR		18.11 ± 7.66^a^			9.06 ± 3.83^a^
T3 - AMF		16.14 ± 7.29^b^			8.07 ± 3.64^b^
T4 - PGPR + AMF		17.84 ± 7.05^a^			8.92 ± 3.53^a^
**Block**		**V (dm**^**3**^**)**			**MAI (dm**^**3**^ **year**^**−1**^**)**
**Tukey test of block**					
1		19.85 ± 7.19^a^			9.92 ± 3.59^a^
2		21.03 ± 6.96^ab^			10.52 ± 3.48^ab^
3		18.50 ± 7.63^b^			9.25 ± 3.82^b^
4		13.11 ± 5.10^c^			6.55 ± 2.55^c^
5		12.72 ± 5.25^c^			6.36 ± 2.62^c^
**Block**	**T1 - Control**	**T2 – PGPR**	**T3 – AMF**		**T4 - PGPR + AMF**
**Tukey test of A × B V (dm**^**3**^**)**					
1	17.27 ± 4.6^cdef^	24.92 ± 6.53^ab^	14.22 ± 6.42^defgh^		23.07 ± 5.75^abc^
2	25.74 ± 5.75^a^	18.87 ± 6.85^bcde^	21.36 ± 7.27 ^abc^		18.55 ± 5.72^cde^
3	16.63 ± 6.23^cdefg^	19.79 ± 7.19 ^abcd^	18.83 ± 8.63 ^bcde^		18.49 ± 8.28^cde^
4	12.90 ± 4.61^efgh^	14.57 ± 5.68^defgh^	10.87 ± 4.49^gh^		14.51 ± 5.23^defgh^
5	8.78 ± 2.89^h^	11.88 ± 4.58^fgh^	15.12 ± 4.24^defgh^		14.22 ± 6.42^defgh^
**MAI (dm**^**3**^ **year**^**−1**^**)**					
1	8.63 ± 2.31^cdef^	12.46 ± 3.26^ab^	7.11 ± 3.21^defgh^		11.53 ± 2.88^abc^
2	12.87 ± 2.87^a^	9.43 ± 3.43^bcde^	10.68 ± 3.63^abc^		9.28 ± 2.86^cde^
3	8.32 ± 3.11^cdefg^	9.89 ± 3.59^abcd^	9.41 ± 4.32^bcde^		9.24 ± 4.14^cde^
4	6.45 ± 2.30^efgh^	7.28 ± 2.84^defgh^	5.43 ± 2.25^gh^		7.26 ± 2.62^defgh^
5	4.39 ± 1.45^h^	5.94 ± 2.29^fgh^	7.56 ± 2.12^defgh^		7.11 ± 3.21^defgh^

* *Significant difference (p ≤ 0.05). Same letter does not differ by the Tukey test (p ≤ 0.05)*.

At 3 years, the V, MAI, and CAI also presented statistical differences between treatments, block, and the interaction between treatment × block. The best treatment was T2, which differed significantly from the control. In the average value of treatments, PGPR (T2) showed an 11.04% increase in V at 3 years followed by PGPR + AMF (T4) with 5.81%, and AMF (T3) with 4.75% compared with the control. Blocks 1, 2, and 3 presented the best results. The interaction of the treatment and blocking factors was significant but did not show any clear pattern. This difference between the blocks may be due to variations in soil conditions, which is due to the large experimental area (Table 9).

**Table 9:** Tree volume, mean annual increment, and current annual increment at 3 years old in plants of *Tectona grandis* in Pará state, Brazil.

**Factor**		**V (dm**^****3****^**)**		**MAI (dm**^****3****^ **year**^****−1****^**)**		**CAI (dm**^****3****^**)**	
	**df**	***F***	***p*-value**	***F***	***p*-value**	***F***	***p*-value**
**ANOVA - Pará, 3 years old**							
Treatment (A)	3	5.237	0.0015^*^	5.237	0.0015^*^	5.299	0.0014^*^
Block (B)	4	27.375	0.0000^*^	27.378	0.0000^*^	20.983	0.0000^*^
A × B	12	7.408	0.0000^*^	7.409	0.0000^*^	6.553	0.0000^*^
Error		903		100.3		712	
**Treatment**		**V (dm**^**3**^**)**		**MAI (dm**^**3**^ **year**^**−1**^**)**		**CAI (dm**^**3**^**)**	
**Tukey test Treatment**							
T1 - Control		128.64 ± 7.37^b^		42.88 ± 3.68^b^		112.14 ± 32.33^b^	
T2 - PGPR		144.61 ± 7.66^a^ (↑ 11.04%)		48.20 ± 3.83^a^		126.50 ± 32.63^a^	
T3 - AMF		135.06 ± 7.29^ab^ (↑ 4.75%)		45.02 ± 3.64^ab^		118.92 ± 27.56^ab^	
T4 - PGPR + AMF		136.58 ± 7.05^ab^ (↑ 5.81%)		45.53 ± 3.53^ab^		118.74 ± 30.50^ab^	
**Block**		**V (dm**^**3**^**)**		**MAI (dm**^**3**^ **year**^**−1**^**)**		**CAI (dm**^**3**^**)**	
**Tukey test of block**							
1		152.98 ± 33.19^a^		50.99 ± 11.06^a^		133.13 ± 29.78^a^	
2		146.56 ± 36.06^a^		48.85 ± 12.02^a^		125.52± 31.70^a^	
3		143.68 ± 36.70^a^		47.89 ± 12.23^a^		125.18± 31.45^a^	
4		117.69 ± 23.26^b^		39.23 ± 7.75^b^		104.58± 20.78^b^	
5		118.19 ± 33.14^b^		39.40 ± 11.05^b^		105.47± 29.22^b^	
**Block**	**T1 - Control**		**T2 – PGPR**		**T3 – AMF**		**T4 - PGPR + AMF**	
**Tukey test of A × B V (dm**^**3**^**)**							
1	144.48 ± 19.32^abcde^		170.65 ± 41.99^a^		133.10 ± 25.54^bcdef^		163.96 ± 29.83^ab^	
2	165.48 ± 34.14^a^		143.06 ± 36.93^abcde^		154.97 ± 33.49^abc^		123.48 ± 26.81^def^	
3	129.35 ± 27.49^cdef^		152.70 ± 39.41^abcd^		147.54 ± 39.46^abcde^		143.16 ± 36.65^abcde^	
4	117.26 ± 22.21^ef^		129.59 ± 25.38^cdef^		109.18 ± 19.69^fg^		118.02 ± 23.81^ef^	
5	76.01 ± 20.12^g^		124.68 ± 20.44^cdef^		129.30 ± 20.50^cdef^		132.57 ± 37.17^cdef^	
**MAI (dm**^**3**^ **year**^**−1**^**)**							
1	48.16 ± 6.44^abcde^		56.88 ± 14.00^a^		44.37 ± 8.51^bcdef^		54.65 ± 9.94^ab^	
2	55.16 ± 11.38^a^		47.69 ± 12.31^abcde^		51.66 ± 11.16^abc^		41.16 ± 8.94^def^	
3	43.12 ± 9.16^cdef^		50.90 ± 13.14^abcd^		49.18 ± 13.15^abcde^		47.72 ± 12.22^abcde^	
4	39.09 ± 7.40^ef^		43.20 ± 8.46^cdef^		36.39 ± 6.56^fg^		39.34 ± 7.94^ef^	
5	25.34 ± 6.71^g^		41.56 ± 6.81^cdef^		43.10 ± 6.83^cdef^		44.19 ± 12.39^cdef^	
**CAI (dm**^**3**^**)**							
1	127.22 ± 17.71^abcde^		145.73 ± 38.71^a^		118.88 ± 25.01^abcdef^		140.89 ± 28.35^ab^	
2	139.74 ± 30.14^abc^		124.19 ± 32.95^abcdef^		133.61 ± 28.87^abc^		104.93 ± 24.63^def^	
3	112.72 ± 24.56^cdef^		132.92 ± 34.26^abcd^		128.71 ± 33.35^abcde^		124.68 ± 30.60^abcdef^	
4	104.35 ± 18.84^ef^		115.02 ± 25.78^abcdef^		98.31 ± 16.70^f^		103.51 ± 20.97^ef^	
5	67.23 ± 18.76^g^		112.80 ± 17.22^cdef^		114.18 ± 17.75^bcdef^		118.36 ± 31.87^abcdef^	

* *Significant difference (p ≤ 0.05). Same letter does not differ by the Tukey test (p ≤ 0.05)*.

*↑Percentage of gain in relation to the control treatment*.

## Discussion

The PGPR (*Bacillus subtilis*) inoculation and co-inoculation with AMF caused an increase in seedlings survival. In this period, the survival of seedling is directly related with the increase in cuttings rooting. *B subtilis* can assist in this process through the phytohormone production of indoleacetic acid (IAA) (Radhakrishnan et al., 2017). According to Teixeira et al. (2007), the mini cuttings of eucalypt clones in substrate with inoculation of *B subtilis* shows better root formation, and this result allows optimization of seedling development in clonal nurseries. The presence of rhizobacteria *B. subtilis* can directly influence plant growth not only by phytohormone production (Ali et al., 2009; Galaviz et al., 2018) but also by stress-inhibiting enzymes, siderophores, and indirect P-solubilization (Meng et al., 2016).

Root colonization by AMF *Rhizophagus clarus* was around 60% at 90 days after planting. The AMF efficiency must be evaluated with data of vegetative development, since symbiosis may not be effective, with high colonization rates, depending on the host. Arbuscular mycorrhizal (AM) association can be influenced and differentiated by vegetal species, clones, and cultivars, as well as by inoculated AMF species (Smith and Smith, 2011). In an evaluation of Indian native AMF species (*Funneliformis mosseae, Glomus intraradices*, and *Glomus proliferum*), Ajeesh et al. (2017) observed that colonization range was from 15 to 36%, and that teak response was better with *G proliferum* than with other mycorrhizal species. In Indonesia, a study on *Acaulospora* sp., *Gigaspora* sp., and mixed *Acaulospora* sp. and *Gigaspora* sp, combined with different doses of compost in soil, showed that seedlings inoculated with *Gigaspora* sp. and 15% of compost increased seedling quality, and the root colonization of teak in this study ranged from 0 to 36% for *Acaulospora* sp and from 32 to 74% for *Gigaspora* sp (Prayudyaningsih and Sari, 2016).

AMF colonization response also can be modulated by the successional status of plant host. *Tectona grandis* has been reported as an early successional species (Chen et al., 2011). Early successional woody species with high metabolic rate dominate the initial stages of succession, occur in highly illuminated environments, and usually exhibit high mycorrhizal colonization and responsiveness. In contrast, late-successional woody species with low metabolic rate dominant in mature forest occur in an environment with low light incidence and exhibit low mycorrhizal colonization and responsiveness (Zangaro et al., 2007). High mycorrhizal colonization and responsiveness or early successional species can be modified when these are propagated in fertile substrates, leading to modification of the root architecture by the increase in root density and specific root length.

Biometric evaluation in clonal seedlings showed that *R clarus* colonization induced a differential response in growth parameters. *R clarus* had no negative effect on the growth of aerial parts of teak. It also did not significantly affect biomass and branching of the root system. Stem diameter was decreased, and root branching stimulated the co-inoculated PGPR + AMF treatment (Figure 3). One of the factors that lead to this response can be the substrate fertility and container volume used in nurseries. Some authors, such as Zangaro et al. (2015), have described a similar response in seedlings inoculated with AMF in 50 cm^3^ tubes and high fertility substrates, where plants showed growth depression. The depression was possibly related to the AMF association cost for the host plant, especially related to carbon; AMF are dependent on organic carbon from plant photosynthates, and the benefits of plant-fungus association can be affected by the decrease in the photosynthetic rate of environments with light limitation (Smith and Read, 2008). In this context, the carbon cost of AM overcomes the benefits of mineral absorption for the plant. Therefore, the allocation of resources for the maintenance of AMF can be advantageous for the plant, although it does not result in large accumulation of vegetative biomass, as the nutrients acquired *via* AMF can be allocated to increase the growth capacity of the plants in the field.

The nutrients in the foliar analysis for the nursery phase correlated positively with the treatments with microorganisms, in contrast to the control treatment, which did not present any correlation. The chemical properties of soils can be limiting for teak growth (Jerez-Rico and Coutinho, 2017). Well-nourished seedlings can be fundamental for good development in the field.

In addition to well-nourished seedlings in the field, the presence of PGPR and AMF can bring several benefits. For example, teak is demanding the availability of nutrients, such as nitrogen, phosphorus, potassium, calcium, and magnesium. Base saturation should be >50%, high pH (>5.5) and low aluminum presence (Matricardi, 1989; Jerez-Rico and Coutinho, 2017). The AMF have been reported to increase the tolerance of teak trees to acidity or high concentrations of Al (Alvarado et al., 2004), and PGPR can be acting in P-solubilization (Meng et al., 2016), helping the plants not to suffer in poor soils such as the soils in the planting areas of this experiment.

The beneficial approach of microorganism inoculation for the development of woody plants in fields has been reported (Siviero et al., 2008; Cely et al., 2016; Duin et al., 2019). Root colonization by AMF in teak (*T grandis* L.f.) has been evaluated by other authors, such as Irianto and Santoso (2005). In this study, the inoculation of *Glomus aggregatum* and a mixture containing *Gigaspora margarita, Glomus manihotis, Glomus etunicatum*, and *Acaulospora spinosa* accelerated the height and diameter growth by 61 and 47%, respectively, after 3 months in the field. In our study, in the Pará state, plant growth was positively affected by PGPR in both second and third year and by PGPR + AMF in the third year with the increase in V by 11.04% (PGPR) followed by PGPR + AMF with an increase of 5.81% compared with the control (Tables 6, 7).

The potential of the inoculation of microorganisms for teak development in the nursery and field was recently shown by Raghu et al. (2020), showing that the inoculation of a microbial consortium (*A leptotich*a + *A chroococcum* + *T harzianum*) can improve the growth of plants 289% more than uninoculated plants. These responses of growth promotion can vary depending on the species of microorganisms inoculated, climate characteristics in the field, and genetic profile of teak cultivars, as observed in the evaluations at 2 and 3 years for tree volume (V), mean annual increment (MAI), and current annual increment (CAI).

Thus, a combination of bio-based products in the production of teak seedlings can provide quality seedlings that have good results in the field, even on nutritionally poor soils. The adoption of the use of commercial bio-based products helps to implement this technology in the routine of teak-producing nurseries.

## Data Availability Statement

The original contributions presented in the study are included in the article/supplementary materials, further inquiries can be directed to the corresponding author/s.

## Author Contributions

FS, LV, and IH: coordination, execution, monitoring of experiments, and data processing. DS, AP, MB, BF, DD, AC, and AS: laboratory and field data collection. BG, ML, and GA: arbuscular mycorrhizal inoculum production, scientific support, and manuscript writing. RF and MT: idealization, coordination, and project planning. ES, JB, and FT: coordination, technical, and financial support at the Teak Resources Company (TRC). All authors contributed to the article and approved the submitted version.

## Conflict of Interest

JB, ES, and FT are employed by the Teak Resources Company (TRC). The remaining authors declare that the research was conducted in the absence of any commercial or financial relationships that could be construed as a potential conflict of interest.

## References

[B1] Ajeesh R. Santhoshkuma A.V. Go S. (2017). Screening of selected native arbuscular mycorrhizal fungi at different levels for their symbiotic efficiency with *Tectona grandis* seedlings. J. Trop. For. Sci. 29, 395–403. 10.26525/jtfs2017.29.4.395403

[B2] Akinrinlola R. J. Yuen G. Y. Drijber R. A. Adesemoye A. O. (2018). Evaluation of *Bacillus* strains for plant growth promotion and predictability of efficacy by *in vitro* physiological traits. Int. J Microb. 2018:5686874. 10.1155/2018/5686874 30402105 PMC6192143

[B3] Alam M. Khaliq A. Sattar A. Shukla R.S. Anwar M. Seema Dharni S. (2011). Synergistic effect of arbuscular mycorrhizal fungi and *Bacillus subtilis* on the biomass and essential oil yield of rose-scented geranium (*Pelargonium graveolens*). Arch. Agron. Soil. Sci. 57, 889–898. 10.1080/03650340.2010.498013

[B4] Ali B. Sabri A. N. Ljung K. Hasnain S. (2009). Auxin production by plant associated bacteria: impact on endogenous IAA content and growth of *Triticum aestivum* L. Lett. Appl. Microbiol. 48, 542–547. 10.1111/j.1472-765X.2009.02565.x 19220737

[B5] Alvarado A. Chavarría M. Guerrero R. Boniche J. Navarro J. R. (2004). Características edáficas y presencia de micorrizas en plantaciones de teca (*Tectona grandis*L.f.) en Costa Rica. AgronomíaCostarricense. 28, 89–100. Available online at: https://www.redalyc.org/articulo.oa?id=43628109

[B6] Andrade G. Cely M.V.T. (2019). Processo de Produção e Inoculação de Fungos Micorrízicos Arbusculares. BR Patent No 102019008109-0 A2.

[B7] Awasthi A. Bharti N. Nair P. Singh R. Shukla A. Gupta M.. (2011). Synergistic effect of *Glomus mosseae* and nitrogen fixing *Bacillus subtilis* strain Daz26 on artemisin content in *Artemisia annua* L. Appl. Soil Ecol. 49, 125–130. 10.1016/j.apsoil.2011.06.005

[B8] Carneiro M. F. Albuquerque W. W. Souza C. O. Câmara A. P. Silva J. G. M. Galvão E. K. S. (2018). Radial growth and artificial form factor of teak trees in Alta Floresta do Oeste, Rondônia. Rev. Cienc. Agrar. 61, 1–7. 10.22491/rca.2018.2702

[B9] Cely M. V. T. Siviero M. A. Emiliano J. Spago F.R. Freitas V. F. Barazetti A. R.. (2016). Inoculation of *Schizolobiumparahyba* with mycorrhizal fungi and plant growth-promoting rhizobacteria increases wood yield under field conditions. Front. Plant Sci. 7, 1–13. 10.3389/fpls.2016.01708 27920781 PMC5118453

[B10] Chen J.W Zhanga Q. Lia X.S. Cao K.F. (2011). Steady and dynamic photosynthetic responses of seedlings from contrasting successional groups under low-light growth conditions. Physiol. Plantarum. 141, 84–95. 10.1111/j.1399-3054.2010.01414.x 20875058

[B11] Duin V. F. F. Liuti G. Prado N. V. Cely M. V. T. Andreata M. F. L. Santos I. M. O.. (2019). Effect of the fertilization and growth promoting microrganisms on *Schizolobiumparahyba*. Semina Ciênc. Agrár. 40, 1747–1760. 10.5433/1679-0359.2019v40n5p1747

[B12] FAO (1994). Soil Map of the World. Revised Legend With Corrections. Rome: FAO-UNESCOISRIC, 140.

[B13] Galaviz C. Lopez B. R. Bashan L. E. Hirsch A. M. Maymon M. Bashan Y. (2018). Root growth improvement of mesquite seedlings and bacterial rhizosphere and soil community changes are induced by inoculation with plant growth-promoting bacteria and promote restoration of eroded desert soil. Land Degrad. Dev. 2018:2904. 10.1002/ldr.2904

[B14] Giovannetti M. Mosse B. (1980). An evaluation of techniques for measuring vesicular arbuscular mycorrhizal infection in roots. New Phytol. 84, 489–500. 10.1111/j.1469-8137.1980.tb04556.x

[B15] Hashem A. Abd_Allah E. F. Alqarawi A. A. Al-Huqail A. A. Shah M. A. (2016). Induction of osmoregulation and modulation of salt stress in *Acacia gerrardii* Benth. by arbuscular mycorrhizal fungi and *Bacillus subtilis* (BERA 71). Bio. Med. Res. Int. 2016:6294098. 10.1155/2016/6294098 27597969 PMC5002495

[B16] IBÁ. (2019). Relatório IBÁ 2019 – Desempenho do setor florestal em 2018. Brasília.

[B17] Irianto R.S.B. Santoso E. (2005). Effect of arbuscular mycorrhiza fungi inoculation on teak (*Tectona grandis* Linn. *F*) at cikampek, west Java. J. For. Res. 2, 69–73. 10.20886/ijfr.2005.2.2.69-73

[B18] Jerez-Rico M. Coutinho S. A. (2017). Establishment and management of planted teak forests, in The Global Teak Study. Analysis, Evaluation and Future Potential of Teak Resources IUFRO World Series, Vol. 36, eds Kollert W. Kleine M. (Vienna), 49–65.

[B19] Köppen W. (1936). Das geographische System der Klimate, in Handbuch der Klimatologie, Vol. 1, eds Köppen W. Geiger R. (Berlin: Verlag von Gebrüder Borntraeger), 1–44.

[B20] Marulanda-Aguirre A. Azcón R. Ruiz-Lozano J. M. Aroca R. (2007). Differential effects of a *Bacillusmegaterium* Strain on *Lactuca sativa* plant growth depending on the rigin of the arbuscular mycorrhizal fungus co-inoculated: physiologic and biochemical traits. J. Plant. Growth Regul. 27, 10–18. 10.1007/s00344-007-9024-5

[B21] Matricardi W. A. T. (1989). Efeitos dos fatores do solo sobre o desenvolvimento da teca (Tectona grandis L. F.) cultivada na grande Cáceres - Mato Grosso (Dissertação, Mestrado em Ciências Florestais), Universidade de São Paulo, Piracicaba, 135.

[B22] Medina A. Probanza A. Gutierrez Mañero F. J. Azcón R. (2003). Interactions of arbuscular-mycorrhizal fungi and *Bacillus* strains and their effects on plant growth, microbial rhizosphere activity (thymidine and leucine incorporation) and fungal biomass (ergosterol and chitin). App. Soil. Ecol. 22, 15–28. 10.1016/S0929-1393(02)00112-9

[B23] Meng Q. Jiang H. Hao J. J. (2016). Effects of *Bacillus velezensis* strain BAC03 in promoting plant growth. Biol. Control. 98, 18–26. 10.1016/j.biocontrol.2016.03.010

[B24] Meza A. Rodriguez J. Gatti K. Espinoza E. (2015). Propagación de arboles de teca *Tectona grandis* L. f. por miniestacas. Temas Agrar. 20, 43–48. 10.21897/rta.v20i2.757

[B25] Pandey D. Brown C. (2000). Teak: A Global Overview (Unasylva: FAO), 3–13.

[B26] Phillips J. M. Hayman D. S. (1970). Improved procedures for clearing roots and staining parasitic and vesicular-arbuscular mycorrhizal for rapid assessment of infection. Trans. Br. Mycol. Soc. 55, 158–161. 10.1016/S0007-1536(70)80110-3

[B27] Prayudyaningsih R. Sari R. (2016). The application of arbuscular mycorrhizal fungi (AMF) and compost to Improve the growth of Teak seedlings (*Tectona grandi*s Linn. f.) on limestone post-mining soil. J. P. K. Wallacea 5, 37–46. 10.18330/jwallacea.2016.vol5iss1pp37-46

[B28] R Core Team (2020). R: A Language and Environment for Statistical Computing. Vienna: R Foundation for Statistical Computing. Available online at: https://www.R-project.org/ (accessed December 3, 2020).

[B29] Radhakrishnan R. Hashem A. Abd_Allah E. F. (2017). *Bacillus*: a biological tool for crop improvement through bio-molecular changes in adverse environments. Front. Physiol. 8, 1–14. 10.3389/fphys.2017.00667 28932199 PMC5592640

[B30] Raghu H. B. Ashwin R. Ravi J. E. Bagyaraj D. J. (2020). Microbial consortium improved growth and performance of Teak (*Tectona grandis* L.f.) in nursery and field trials. Proc. Natl. Acad. Sci. India Sect. B Biol. Sci. 90, 903–909. 10.1007/s40011-019-01163-0

[B31] Rillig M.C. Mummey D.L. (2006). Mycorrhizas and soil structure. New Phytol. 171, 41–53. 10.1111/j.1469-8137.2006.01750.x 16771981

[B32] Rodrigues L.A. Barroso D.G. Figueredo F.A.M. (2018). Fungos micorrízicos arbusculares no crescimento e na nutrição mineral de mudas de *Tectona grandis* L. F. Ciência Florest. 28, 25–34. 10.5902/1980509831572

[B33] Ryser P. Lambers H. (1995). Root and leaf attributes accounting for the performance of fast- and slow-growing grasses at different nutrient supply. Plant Soil. 170, 251–265. 10.1007/BF00010478

[B34] Schuhli G.S. Paludzyszyn Filho E. (2010). O cenário nacional da silvicultura de teca (*Tectona grandis* L. f.) e perspectivas de melhoramento. Pesqui. Florest. Bras. 30, 217–230. 10.4336/2010.pfb.30.63.217

[B35] Siviero M. A. Marega A. Lima Dos Santos D. Rosselli B. R. Huh Y. S. Santinoni I.. (2008). Interaction among N-fixing bacteria and AM fungi in Amazonian legume tree (*Schizolobiumamazonicum*) in field conditions. Appl. Soil Ecol. 39, 144–152. 10.1016/j.apsoil.2007.12.004

[B36] Smith S. E. Read D. J. (2008). Growth and carbon economy of arbuscular mycorrhizal symbionts, in Mycorrhizal Symbiosis, 3rd Edn, eds Smith S. E. Read D. J. (London: Elseiver), 119–187. 10.1016/B978-012370526-6.50006-4

[B37] Smith S. E. Smith F. A. (2011). Roles of arbuscular mycorrhizas in plant nutrition and growth: new paradigms from cellular to ecosystem scales. Ann. Rev. Plant Biol. 62, 227–250. 10.1146/annurev-arplant-042110-103846 21391813

[B38] Teixeira D. A. Alfenas A. C. Mafia R. G. Ferreira E. M. Siqueira L. Maffia L. A.. (2007). Rhizobacterial promotion of eucalypt rooting and growth. Brazil. J. Microbiol. 38, 118–123. 10.1590/S1517-83822007000100025

[B39] Zangaro W. Nishidate F.R. Vandresen J. Andrade G. Nogueira M.A. (2007). Root mycorrhizal colonization and plant responsiveness are related to root plasticity, soil fertility and successional status of native woody species in southern Brazil. J. Trop. Ecol. 23, 53–62. 10.1017/S0266467406003713

[B40] Zangaro W. Torezan M. D. Rostirola L. V. Souza P. B. Nogueira M. A. (2015). Influence of mycorrhizas, organic substrates and container volumes on the growth of *Heliocarpuspopayanensis* Kunth. Cerne 21, 395–403. 10.1590/01047760201521031335

